# Endothelium-Derived Hyperpolarizing Factor and Myoendothelial Coupling: The *in vivo* Perspective

**DOI:** 10.3389/fphys.2020.602930

**Published:** 2020-12-23

**Authors:** Kjestine Schmidt, Cor de Wit

**Affiliations:** ^1^Institut für Physiologie, Universität zu Lübeck, Lübeck, Germany; ^2^Deutsches Zentrum für Herz-Kreislauf-Forschung (DZHK) e.V. (German Center for Cardiovascular Research), Partner Site Hamburg/Kiel/Lübeck, Lübeck, Germany

**Keywords:** connexins, gap junctions, endothelium-dependent hyperpolarization, myoendothelial coupling, microcirculation

## Abstract

The endothelium controls vascular tone adopting blood flow to tissue needs. It releases chemical mediators [e.g., nitric oxide (NO), prostaglandins (PG)] and exerts appreciable dilation through smooth muscle hyperpolarization, thus termed endothelium-dependent hyperpolarization (EDH). Initially, EDH was attributed to release of a factor, but later it was suggested that smooth muscle hyperpolarization might be derived from radial spread of an initial endothelial hyperpolarization through heterocellular channels coupling these vascular cells. The channels are indeed present and formed by connexins that enrich in gap junctions (GJ). *In vitro* data suggest that myoendothelial coupling underlies EDH-type dilations as evidenced by blocking experiments as well as simultaneous, merely identical membrane potential changes in endothelial and smooth muscle cells (SMCs), which is indicative of coupling through ohmic resistors. However, connexin-deficient animals do not display any attenuation of EDH-type dilations *in vivo*, and endothelial and SMCs exhibit distinct and barely superimposable membrane potential changes exerted by different means *in vivo*. Even if studied in the exact same artery EDH-type dilation exhibits distinct features *in vitro* and *in vivo*: in isometrically mounted vessels, it is rather weak and depends on myoendothelial coupling through connexin40 (Cx40), whereas *in vivo* as well as *in vitro* under isobaric conditions it is powerful and independent of myoendothelial coupling through Cx40. It is concluded that EDH-type dilations are distinct and a significant dependence on myoendothelial coupling *in vitro* does not reflect the situation under physiologic conditions *in vivo*. Myoendothelial coupling may act as a backup mechanism that is uncovered in the absence of the powerful EDH-type response and possibly reflects a situation in a pathophysiologic environment.

## Introduction

**Figure 1:**
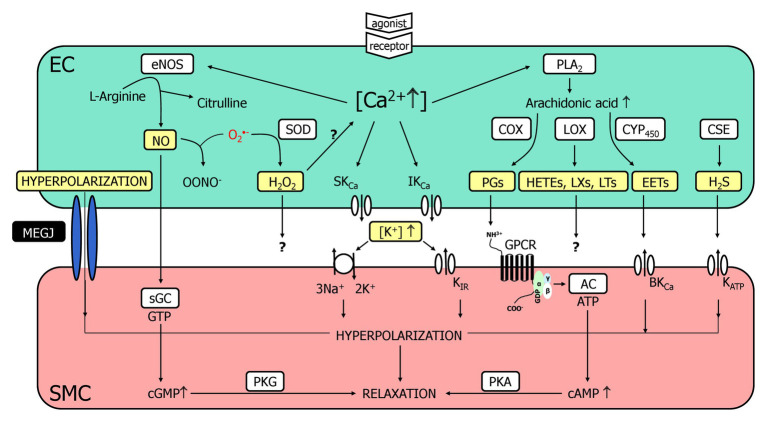
A multitude of endothelial molecules and mechanisms (depicted in yellow boxes) relax vascular smooth muscle. The well-accepted chemical mediators such as nitric oxide (NO) and prostaglandins (PG) are shown, and a variety of substances that have been suggested to underly the endothelium-dependent hyperpolarization (EDH)-type dilation. Importantly, a completely distinct pathway, namely radial electrotonic spread of endothelial hyperpolarizations through myoendothelial coupling was also proposed to initiate the EDH-type dilation. However, the evidence was mostly acquired *in vitro*, whereas data obtained *in vivo* argue against this hypothesis. This justifies reconsideration of other molecules as outlined. For further details, see text. AC, adenylyl cyclase; cAMP, cyclic adenosine monophosphate; cGMP, cyclic guanosine monophosphate; COX, cyclooxygenase; CSE, cystathionine-lyase; CYP450, cytochrome P450 epoxygenase; EC, endothelial cell; eNOS, endothelial nitric oxide synthase; EETs, epoxyeicosatrienoic acids; GPCR, G-protein coupled receptor; HETEs, hydroxyeicosatrienoic acids; H_2_O_2_, hydrogen peroxide; H_2_S, hydrogen sulfide; K_Ca_, Ca^2+^-activated K^+^-channel with small (SK_Ca_), intermediate (IK_Ca_) or big conductance (BK_Ca_); K_ATP_, ATP-sensitive K^+^-channels; K_IR_, inwardly rectifying K^+^-channel; LOX, lipoxygenase; LTs, leukotrienes; LXs, lipoxines; MEGJ, myoendothelial gap junctions; NO, nitric oxide; OONO^−^, peroxynitrite anion; O_2_^−^, superoxide anion; PGs, prostaglandins; PKA, protein kinase A; PKG, protein kinase G; PLA_2_, phospholipase A_2_; sGC, soluble guanylyl cyclase; SMC, smooth muscle cell; SOD; superoxide dismutase.

The endothelium contributes importantly to the regulation of vascular tone and thereby controls organ perfusion. Endothelial cells (ECs) communicate in different languages (or signaling pathways) to smooth muscle cells (SMCs), one of which is the release of chemical substances or mediators that diffuse through the narrow intercellular space before reaching membrane or intracellular proteins as receptors. The outstanding molecules acting as chemical mediators in this pathway of intercellular communication are PG [cyclooxygenase (COX) products] and the gaseous transmitter nitric oxide (NO) which is produced by endothelial NO-synthase and easily diffuses through plasma membranes to reach its target, the intracellularly localized soluble guanylyl cyclase (sGC; Qian and Fulton, 2013; Luo et al., 2016; Nava and Llorens, 2019). Apart from these molecules, other chemical entities have been suggested to be released from ECs and relax smooth muscle, among them K^+^ ions, lipophilic compounds (e.g., epoxyeicosanoids, EETs), proteins (C-type natriuretic peptide), radicals [hydrogen peroxide (H_2_O_2_)], and other gaseous molecules [CO, hydrogen sulfide (H_2_S); Figure 1; Feletou and Vanhoutte, 2009; Garland et al., 2011; Feletou et al., 2012; Ellinsworth et al., 2014, 2016; Feletou, 2016; Freed and Gutterman, 2017; Garland and Dora, 2017].

However, this is a “sticky” business as it is difficult to imagine that several of these compounds are easily and quickly transferable. The smooth muscle relaxes by the opening of K^+^-channels, which induce relaxation likely through closure of voltage-gated Ca^2+^-channels representing a rather speedy signaling pathway. Since the dilaton is depending on the presence of ECs and characterized by smooth muscle hyperpolarization, this obscure mediator has been termed endothelium-derived hyperpolarizing factor (EDHF) assuming that also this mechanism communicates in the same language, i.e., release of a mediator as for the classical autacoids, NO, and PG. However, this view was challenged and a direct electrotonic way of communication was suggested. Charge is transferred from ECs to smooth muscle through intercellular channels [myoendothelial gap junctions (GJs), MEGJs] driven by an initial endothelial hyperpolarization that spreads radially into the vessel wall (Figure 1; de Wit and Griffith, 2010; Freed and Gutterman, 2017; Garland and Dora, 2017).

We describe in this short review the structural requirements and illuminate the experimental evidence obtained *in vivo* that argues against or in favor of the hypothesis that EDHF indeed is an EDH-type dilation rather than being a freely diffusible molecule. Other important functions of myoendothelial coupling, including the transfer of calcium or intracellular messengers, such as inositol-1,4,5-triphosphate (IP3) that modulate vascular responses (Looft-Wilson et al., 2017; Biwer et al., 2018; Wei et al., 2018; Wilson et al., 2019) are reviewed in detail elsewhere (Billaud et al., 2014; Straub et al., 2014; Pogoda and Kameritsch, 2019; Pohl, 2020) and are not addressed in detail in this manuscript. This aspect is mainly examined *in vitro* and due to the lack of experimental data, it is currently unknown if such a signaling pathway is of importance *in vivo*.

## Gap Junctions and the Molecular Bricks (Connexins)

Gap junctions are composed of connexin proteins and six of these molecules assemble to form a hexameric hemichannel. Two of them from adjacent cells dock together and interlink the cytoplasms of these cells through a channel that is tightly sealed against the extracellular environment. The intercellular channels either connect equal (homocellular) or different cell types (heterocellular, e.g., myoendothelial coupling) and allow transfer of small molecules (< 1 kDa) and ions. The latter equals current flow along an electrochemical potential difference and, ultimately, permits the transfer of membrane potential changes between interconnected cells. Hundreds of such channels are located at GJ where adjoining plasma membranes are only 2 nm apart (Saez et al., 2003; Nielsen et al., 2012; Grosely and Sorgen, 2013). GJ can be visualized by electron microscopy and appears as an electron-dense pentalaminar area that can be easily identified between adjacent ECs because of their abundance and the large size of the plaques. They are found to a lesser extent between SMCs, which is possibly due to the smaller plaque size and the limited number of GJ (Sandow et al., 2012). Alternatively, the presence of GJ may be implicated from the demonstration of connexin protein expression by immunostaining. A tremendous amount of publications detected connexins in vascular cells (ECs and SMCs) but this imaging technique does not allow to distinguish homocellular GJ from heterocellular GJ (de Wit, 2004; Figueroa et al., 2004). Moreover, connexin staining is indicated in many cases but the demonstration of a proper functional localization, i.e., inside the plasma membrane, is lacking.

The family of connexin genes is comprised of ~20 members (in humans 21, in mice 20), and the proteins are named according to their theoretical molecular mass; for example, connexin40 (Cx40) has a predicted molecular mass of about 40 kDa (Sohl and Willecke, 2004). In the cardiovascular system, Cx40, Cx37, Cx43, and Cx45 are expressed (Haefliger et al., 2004; Morel, 2014). Their expression pattern is not specific for ECS or SMCs, but a subtype preponderance was found: Cx40 and Cx37 prevail in the endothelium throughout the vascular tree and reportedly Cx43 is also expressed specifically in larger vessels (Gustafsson et al., 2003; Sandow et al., 2003; Rummery and Hill, 2004; de Wit et al., 2006; Haddock et al., 2006; Hakim et al., 2008; Sorensen and Holstein-Rathlou, 2012; Pohl, 2020). Cx43 and Cx45 are the dominant subtypes expressed in SMCs (Hill et al., 2002; Wolfle et al., 2007; Schmidt et al., 2012), but Cx37 was also reported for smooth muscle (Sandow et al., 2003; Alonso et al., 2010a), whereas Cx40 is only found in a very few instances in these cells. Interestingly, Cx40 and Cx37 are also found abundantly in renin producing cells and serve important functions in renin secretion (Wagner and Kurtz, 2013).

Having the expression pattern in mind, MEGJ may be composed of Cx40, Cx37, and Cx43 provided from the endothelial side and Cx43, Cx45, and Cx37 from smooth muscle (Sandow et al., 2009, 2012). This important information is experimentally difficult to obtain in vessels, however, in an *in vitro* co-culture system, myoendothelial junctions were composed of Cx40 and Cx43 provided by both cell types. This occured despite the expression of Cx37 in endothelial and SMCs in this artificial system but this connexin was excluded from these junctions (Isakson and Duling, 2005). Experimental data from intact vessels are scarce: in rat basilar artery using serial section and immunoelectron microscopy, Haddock et al. (2006) demonstrated myoendothelial junctions in which Cx40 and Cx37 were located, others confirmed the presence of Cx37 and Cx40 in different vessels (Mather et al., 2005; Isakson et al., 2008), and also Cx43 was identified at MEGJ (Straub et al., 2010; Pogoda et al., 2014).

Recently, Cx37 was shown to be enriched specifically at sites where endothelial and SMCs come into contact (the internal elastic lamina) in murine small skeletal muscle arteries. Functionally, the authors observed in a culture system, a modulation of Cx37 permeability by NO specifically for this connexin (compared to Cx40 and Cx43) and conclude from these findings that NO exerts a specific modulatory effect on myoendothelial junctions due to the fact that Cx37 is the main Cx at this strategic location, and NO preferentially affects its function (Pogoda et al., 2014). The inhibitory effect of NO on Ca^2+^ permeability of MEGJ was elicited through enhanced phosphorylation of Cx37 at position 332 (Y332) by inhibition of the protein tyrosine phosphatase SHP-2. This effect prevented in isolated vessels the Ca^2+^ drainage from the endothelium into the smooth muscle and enhanced thereby Ca^2+^ levels in ECs promoting vasodilation induced by vasoactive agonists (Pogoda et al., 2017). Taken together, these data are somewhat contradictory (Cx37) and can be only regarded as an initial step on the bumpy road with many experimental obstacles to uncover the subtypes of Cx being localized at the MEGJ.

## Experimental Observations Suggesting Electrical Myoendothelial Coupling *in vitro*

Direct injection of electrical current into ECs in isolated arterioles evoked endothelial hyperpolarizations that were conducted electrotonically (i.e., passively) to subjacent SMCs and, conversely, action potentials originating in the arterial media were rapidly conducted to the endothelium (Emerson and Segal, 2000, 2001; Yamamoto et al., 2001). The signal amplitude was reduced in either direction by about 15%, without change in form, suggesting that myoendothelial junctions behave as ohmic resistors that mediate the heterocellular spread of membrane potential changes without rectification (Yamamoto et al., 2001). Circumferential and subsequent longitudinal spread of hyperpolarization was also demonstrated in conduit vessels (strips of rabbit iliac and porcine coronary artery), from which the endothelium had been partly removed, following hyperpolarization of the residual endothelium (Griffith et al., 2002; Selemidis and Cocks, 2007). A direct heterocellular communication invoking larger molecules was visualized using dyes that diffused from initially loaded ECs into SMCs in the media *via* GJs (Griffith et al., 2002; Kansui et al., 2008; Saliez et al., 2008; Billaud et al., 2009).

If such molecules are able to diffuse through GJ it opens the possibility that non-electrotonic mechanisms contribute to vascular tone regulation, e.g., diffusion of IP_3_ and/or Ca^2+^ ions in either direction (Isakson et al., 2007; Isakson, 2008). For example, the transfer of Ca^2+^ from activated smooth muscle to endothelium has been implicated in a feedback mechanism that evokes an endothelial dilation, thereby counterbalancing the constriction initiated in the smooth muscle by e.g., phenylephrine (Dora et al., 1997; Yashiro and Duling, 2000; Kerr et al., 2012; Biwer et al., 2018; Wei et al., 2018; Wilson et al., 2019). This feedback is tightly controlled (Straub et al., 2012; Biwer et al., 2018) and, interestingly, the artificial modulation of heterocellular contact in larger arteries may alter their vascular function (Shu et al., 2019). The hypothesis that myoendothelial junctions serve as conduits for charge transfer and represent mechanistically the EDH-type dilation instead of an EDHF that traverses the extracellular space has been reviewed excellently in more detail elsewhere (Griffith, 2004; Figueroa and Duling, 2009; de Wit and Griffith, 2010; Garland et al., 2011; Ellinsworth et al., 2014; Freed and Gutterman, 2017; Garland and Dora, 2017).

Proof of concept additionally requires blockage of the gap junctional pathway and the abrogation of EDH-type responses. However, GJs are difficult to block efficiently and comcomitantly avoid nonspecific effects. Application of short interfering peptides (so-called connexin-mimetic peptides) is a useful strategy that provides even certain specificity for different connexin subtypes depending on the amino acid sequences of the peptide. In fact, numerous studies have shown that they are able to suppress EDHF-type smooth muscle hyperpolarizations and dilations *in vitro* (Griffith, 2004; de Wit and Griffith, 2010). In some vessels, connexin-mimetic peptides targeted against Cx37 and/or Cx40 attenuated endothelium-dependent smooth muscle hyperpolarization (Chaytor et al., 2005), whereas in other arteries, peptide combinations to target Cx37, Cx40, and Cx43 collectively were required to block of EDH-type responses (Chaytor et al., 2001; Matchkov et al., 2006).

Aiming at the protein structure with a blocking antibody provides a distinct, very specific approach to verify the functional necessity of connexins. Mather et al. (2005) loaded ECs with antibodies targeted against the cytoplasmic tail of Cx40 and observed an abrogation of EDH-type responses in rat mesenteric small arteries. This occurred without affecting increases in endothelial calcium and, importantly, antibodies directed against Cx37 and Cx43 were inactive suggesting that Cx40 is a key player in the response (Mather et al., 2005). The endothelium opposes myogenic constrictions through NO release (de Wit et al., 1998) and EDH-type dilations possibly through Ca^2+^ transfer from smooth muscle to endothelium as outlined above. The latter opposing mechanism was abrogated in mice carrying a mutant Cx40 protein, which also impaired chemical coupling (Chaston et al., 2013).

Taken together, there is compelling evidence that myoendothelial coupling through connexins provides a functional pathway that allows electrotonic spread of membrane potential changes (and signaling molecules). Blockade of this pathway attenuates or even abrogates EDH-type dilations *in vitro*. Specifically, Cx40 (and Cx37) seem to be invoked and appear to be a required brick of the intercellular signaling pathway.

## Myoendothelial Coupling as the EDH-Mechanism *in vivo*: The Evidence is Lacking

Collecting evidence *in vivo* is more cumbersome; however, an efficient strategy is the use of mice deficient for connexins. We published the first study on vascular connexin function by using Cx40-deficient mice in which we demonstrated a crucial role for Cx40 to conduct endothelium-dependent dilations longitudinally along the vessel wall, thereby coordinating vascular cellular behavior (de Wit et al., 2000; Wolfle et al., 2007) that was shortly later confirmed by Figueroa et al. (2003). However, Cx40 is not required for radial electrotonic signal transmission because acetylcholine-induced EDH-type dilations were nearly preserved in skeletal muscle arterioles studied by intravital microscopy in anaesthestized Cx40-deficent mice. Later, we re-examined these responses in more detail and demonstrated a very minor (if any) attenuation of EDH-type dilations upon acetylcholine (Milkau et al., 2010).

In awake Cx40-deficient mice, intraarterial acetylcholine reduced blood pressure to the same absolute level as in wildtype controls, despite their initially elevated arterial pressure (de Wit et al., 2003). This hypotensive response can be attributed to the EDHF phenomenon because it is unaffected by NO-synthase inhibition and is preserved in mice with targeted disruption of the NO-cyclic guanosine monophosphate (cGMP) pathway (Koeppen et al., 2004). Furthermore, resistance changes in the renal circulation in response to acetylcholine were comparable in wildtype and Cx40-deficient mice (Just et al., 2009).

Collectively, these results argue against the hypothesis that Cx40 is universally required for EDH-type signaling *in vivo*. These differences may relate indeed due to *in vivo* vs. *in vitro* vascular behavior or caused by different vessel sizes. Arteries studied *in vitro* are usually larger (> 100 μm) than arterioles accessible for intravital microscopy examined *in vivo* (20–50 μm). The latter arterioles are those that provide the vascular resistance, and their behavior is revealed by pressure drop or resistance measurements in intact organs or animals.

To exclude effects related to vessel size differences, we performed measurements on a small murine artery (gracilis artery) that exhibits a diameter (~130 μm) allowing *in vitro* as well as *in vivo* examination because the artery is located at a spot that is accessible for intravital microscopy. This small artery exhibits *in vitro* an acetylcholine-induced dilation that is mediated to a considerable part by an EDH-type mechanism (~60%, the remaining portion is mediated by NO and PG). Thus, the EDH-type dilation is larger than in a conducting artery (femoral artery), which relies mostly on NO but it is smaller than in arterioles. Arteriolar responses in the microcirculation *in vivo* are nearly exclusively mediated by EDH-type dilation (Si et al., 2006; Brahler et al., 2009; Wolfle et al., 2009).

**Figure 2:**
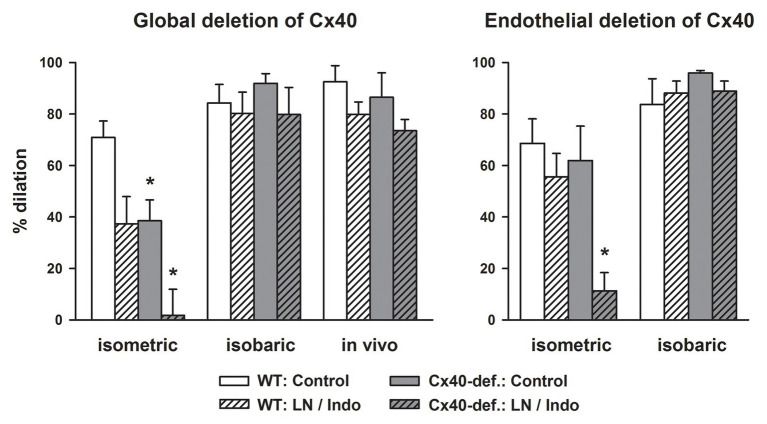
EDH-type dilations are abrogated in gracilis arteries in the absence of connexin40 (Cx40) only if studied *in vitro* in an isometric setting. Acetylcholine (3 μM) induced dilations in gracilis artery studied *in vitro* either in a wire- (isometric) or a pressure-myograph (isobaric) or *in vivo* in anesthetized mice. Cx40 was either deleted globally (left) or in an endothelial-specific manner (right) and compared to respective wildtype mice. Under isometric conditions, blockade of NO-synthase and COX reduced the response in wildtype and abrogated it in Cx40-deficient mice demonstrating a dependence of EDH-type dilations on Cx40 and supposedly on myoendothelial coupling in this setting. In marked contrast, EDH-type dilations were completely preserved in Cx40-deficient mice under isobaric conditions and *in vivo*. Thus, under these conditions, EDH-type dilations are independent of the presence of Cx40. Interestingly, EDH was more powerful under these experimental conditions and induced near full dilation in all genotypes. ^*^ indicates *p* < 0.05 vs. wildtype; LN/Indo, L-nitro-arginine (300 μM) and indometacin (3 μM) to block NO-synthase and COX; Cx40-def, Cx40-deficient mice either global or endothelial-cell specific deletion using Cre recombinase driven by TIE2 in mice carrying homozygously a Cx40 floxed gene. The full data set is published (Boettcher and de Wit, 2011).

In any case, acetylcholine-induced dilations were attenuated in gracilis arteries harvested from Cx40-deficient mice compared to wildtype mice if studied isometrically *in vitro* using wire myography. Blockade of NO-synthase and COX nearly abrogated the dilation in Cx40-deficient arteries but uncovered EDH-type dilations in wildtype mice. This demonstrates that EDH-type dilations require Cx40 in this setting and suggest that myoendothelial coupling requiring Cx40 supports this pathway (Figure 2, left; Boettcher and de Wit, 2011). However, if the exact same vessel was examined at isobaric conditions (pressure myograph), the dilation upon acetylcholine was firstly not attenuated by NO-synthase and COX inhibition in wildtype and secondly fully preserved in Cx40-deficient vessels. This demonstrates that EDH-type dilations, firstly, are more powerful at these experimental conditions (isobaric study) and, secondly, are fully preserved in the absence of Cx40 and, thus, most likely independent of myoendothelial coupling. The exact same results were found if this vessel was studied in the anesthetized mouse *in vivo* (Figure 2, left panel; Boettcher and de Wit, 2011).

In line with this, an attenuation of the EDH-type dilation in Cx40-deficient vessels was also observed in renal arteries (interlobar vessels) that were studied isometrically *in vitro* using wire myography. In addition to myoendothelial coupling, endothelial K^+^ release contributed to the EDH-type dilation in these vessels since a blockade of inwardly rectifying K^+^-channel (K_IR_) and the sodium pump reduced the responses in both, wildtype and Cx40-deficient mice (Brasen et al., 2018). Although the responses were not studied *in vivo* in this study, acetylcholine infusion decreased vascular resistance in the renal circulation measured *in vivo* to a comparable degree in wildtype and Cx40-deficient mice (Just et al., 2009) indicating again intact EDH-type dilations in Cx40-deficient mice *in vivo*.

## Distinct Vascular Responses are not Related to Concomitant Hypertension

Since mice globally deficient for Cx40 are hypertensive due to an abrogated feedback of pressure on renin release (Wagner et al., 2007, 2010; Schweda et al., 2009), hypertension might be a causative factor in impairing the EDH-type dilation through modifying myoendothelial junctions by an alteration of connexin expression (Figueroa et al., 2006; Meens et al., 2013). Therefore, we examined arteries from mice with EC-specific deletion of Cx40, which are normotensive (Wagner et al., 2010; Jobs et al., 2012). However, arteries exhibited similar characteristics: The acetylcholine-induced dilation in isometrically mounted vessels was abrogated after the blockade of NO synthase and COX in the absence of endothelial Cx40 but not in wildtype vessels. This confirmed an EDH-type dilation in wildtype vessels that is not found in the absence of endothelial Cx40 and indicates that this part of the acetylcholine-response is dependent on myoendothelial coupling in this setting.

In marked contrast, a fully intact EDH-type dilation upon acetylcholine was found in vessels from endothelial-specific Cx40-deficient mice if mounted isobarically (Figure 2, right; Boettcher and de Wit, 2011). As before, the EDH-type dilation in the isobaric setting was more powerful in both genotypes, and an attenuation of the response after NO synthase and COX inhibition was neither observed in wildtype vessels nor in vessels deficient for endothelial Cx40 (Figure 2, right). This suggests that this EDH-type dilation, which was only observed in the isobaric setting or *in vivo*, is independent of Cx40. Furthermore, this powerful response is even capable to replace the dilatory function of other endothelial mediators (mainly NO). Importantly, EC-specific Cx40-deficient mice are not hypertensive (Wagner et al., 2010; Jobs et al., 2012) excluding that hypertension modulates connexin expression and that such an alteration had caused the defects observed in globally Cx40-deficient mice.

The divergent results observed in isobaric or isometric vessel study are striking. A key experimental difference is the change in wall tension during constriction and dilation. In isometric conditions, wall tension decreases during dilations and rises during constriction whereas the opposite is true for isobaric (and *in vivo*) conditions. These different experimental setups have also shown to modulate the sensitivity of rat mesenteric arteries for vasoconstrictors (Buus et al., 1994). This may be related to changes in membrane potential because a small initial depolarization did offset the differences in agonist sensitivity (Dunn et al., 1994). Modulatory effects of experimental conditions were also demonstrated on EDH-type dilations: Lowering the extracellular Ca^2+^ concentration shifted the type of endothelial K_Ca_ channel initiating the EDH response from K_Ca_2.3 to K_Ca_3.1. This also affected the link to the vessel relaxation, which shifted from myoendothelial coupling to the activation of the Na^+^/K^+^ ATPase, thus defining distinct EDHF pathways depending on experimental conditions (Dora et al., 2008).

Since ECs express both connexins, Cx40 and Cx37, in abundant amounts, the question arises why Cx37 is not able to replace the function of Cx40. Contrary to an anticipated compensatory upregulation in Cx40-deficient vessels, Cx37 expression is actually downregulated in the aortic endothelium (Simon and McWhorter, 2003), and Cx37 cannot be detected by immunostaining in arterioles (de Wit, 2010; Jobs et al., 2012). Recent work identified spare Cx37 expression in skeletal muscle arterioles and, in addition, demonstrated the presence of myoendothelial junctions in Cx40-deficient mice (Howitt et al., 2013). Interestingly, Cx40 and Cx37 are located tightly together suggesting physical interaction (Alonso et al., 2010b). Thus, Cx40 seems to be required to locate Cx37 appropriately in the endothelial plasma membrane, and a potentially remaining Cx37 expression is functionally insufficient. Conversely, deficiency of Cx37 did not result in noticeable defects of vascular regulation (Figueroa and Duling, 2008) or renin secretion (Wagner et al., 2009) suggesting that the role of Cx37 can be replaced by Cx40.

## EDH-Type Dilations Elicited by Other Agonists are Likewise Independent of Cx40 *in vivo*

Acetylcholine activates muscarinic G-protein coupled receptors (GPCRs) that initiate a Ca^2+^-dependent signaling pathway ultimately leading to endothelial hyperpolarization by means of the activation of endothelial Ca^2+^-dependent K^+^-channels (K_Ca_3.1, K_Ca_2.3; Brahler et al., 2009). This last element in the cascade can be activated pharmacologically by compounds, such as SKA-31 (Sankaranarayanan et al., 2009). In fact, SKA-31 exhibits a higher sensitivity toward K_Ca_3.1 and produces arteriolar dilation *in vivo* specifically *via* activation of this endothelial ion channel and the ensuing endothelial hyperpolarization supposedly occurs without increases of endothelial Ca^2+^. This even more “pure” EDH-type dilation is likewise unimpeded in Cx40-deficient arterioles examined in the microcirculation *in vivo* (Radtke et al., 2013). Similarly, the arterial pressure decreases upon systemic application of SKA-31 were not attenuated in either global or endothelial-specific Cx40-deficient mice (Radtke et al., 2013). This verifies that endothelial hyperpolarization through activation of K_Ca_3.1 initiates an EDH-type dilation which is independent of Cx40 *in vivo* and thus likely independent of myoendothelial junctions.

Taken together, a strong powerful EDH-type dilation exists *in vivo* that acts independent of Cx40. We suggest that we need to reconsider the “sticky” business, which may not be so sticky at all in search for this mediator (Figure 1). Importantly, this powerful mechanism is lost *in vitro* in certain experimental settings (isometric setup). At such conditions, myoendothelial coupling may take over and provide a remaining, less powerful EDH-type dilation that is, in fact, dependent on Cx40.

## Lack of Evidence for Charge Transmission by Myoendothelial Coupling *in vivo*

Having provided evidence that lack of Cx40 (and Cx37) leaves EDH-type dilations fully intact *in vivo* it remains to be considered if myoendothelial coupling exists *in vivo* at all. It may well be that heterocellular coupling is turned off during physiologic vascular control and is only activated during special (pathophysiologic?) conditions. In contrast to the findings with isolated arteries and arterioles described above, a number of studies have failed to demonstrate tight electric myoendothelial coupling in arterioles *in vivo* (de Wit et al., 2008). For example, electrophysiological measurements *in vivo* demonstrated differences in the resting membrane potential of endothelial and SMCs in the order of 10 mV in murine skeletal muscle arterioles (Siegl et al., 2005). Additionally, acetylcholine-induced hyperpolarization of SMCs was selectively blocked by a specific K^+^-channel blocker without modifying endothelial hyperpolarization (Siegl et al., 2005). Conversely, adenosine dilated arterioles by activating smooth muscle K_ATP_ channels but did not hyperpolarize the endothelium (de Wit, 2010); whereas in isolated conduit arteries, K_ATP_ mediated hyperpolarization is transmitted to the endothelium *via* MEGJ (Murai et al., 1999). Also in hamster arterioles *in vivo*, smooth muscle depolarizations did not affect the membrane potential of ECs: hyperpolarizations initiated by acetylcholine differed in amplitude and form between endothelial and SMCs (Welsh and Segal, 1998). Again, endothelial and smooth muscle hyperpolarization exhibited differential sensitivity to inhibitors (Welsh and Segal, 2000). Finally, selective destruction of endothelial or SMCs along the conduction pathway suggests that hyperpolarization is not freely transferred between the two cell types (Budel et al., 2003).

These experimental findings do not provide evidence for functional myoendothelial coupling that aligns the membrane potential of endothelial and SMCs *in vivo*. The data rather suggest that the membrane potential of these distinct cell populations is regulated independently reflecting their specific functions. Interestingly, connexins at the myoendothelial junctions are subject to functional regulation (Straub et al., 2010; Westcott and Segal, 2013), which may provide an explanation for the discrepant *in vivo* findings. Although significant charge transfer through MEGJ was not convincingly demonstrated *in vivo*, significant contributions of myoendothelial coupling to vascular function may well be of importance, for example the transfer of intracellular messengers (Looft-Wilson et al., 2017; Biwer et al., 2018; Wei et al., 2018; Wilson et al., 2019; Pohl, 2020).

## Conclusion

Many *in vitro* studies demonstrate an important role for myoendothelial coupling by transferring charge from endothelial to SMCs to elicit EDH-type dilations. However, *in vivo* a distinct, more powerful mechanism is responsible for such dilations that easily overcome the lack of myoendothelial coupling. The evidence for efficient charge transfer through myoendothelial coupling *in vivo* is scarce. This is not related to a lack of data, in fact, many laboratories have attempted to tackle this important question, but failed to find solid evidence documenting myoendothelial coupling. In fact, these efforts have accumulated strong indications for the opposite, i.e., endothelial and SMCs “do their own thing” with respect to membrane potential. However, under certain experimental conditions, myoendothelial coupling supports EDH-type dilation, but this EDH-type response is rather weak compared to the EDH-mechanism acting *in vivo*.

## Author Contributions

All authors listed have made a substantial, direct and intellectual contribution to the work, and approved it for publication.

### Conflict of Interest

The authors declare that the research was conducted in the absence of any commercial or financial relationships that could be construed as a potential conflict of interest.
